# Melatonin in Wine and Beer: Beneficial Effects

**DOI:** 10.3390/molecules26020343

**Published:** 2021-01-11

**Authors:** Javier Marhuenda, Débora Villaño, Raúl Arcusa, Pilar Zafrilla

**Affiliations:** Department of Pharmacy, Faculty of Health Sciences, Campus de los Jerónimos, Catholic University of San Antonio (UCAM), Guadalupe, 30107 Murcia, Spain; dvillano@ucam.edu (D.V.); rarcusa@ucam.edu (R.A.); mpzafrilla@ucam.edu (P.Z.)

**Keywords:** melatonin, wine, beer, polyphenols, free radical

## Abstract

Melatonin is a hormone secreted in the pineal gland with several functions, especially regulation of circadian sleep cycle and the biological processes related to it. This review evaluates the bioavailability of melatonin and resulting metabolites, the presence of melatonin in wine and beer and factors that influence it, and finally the different benefits related to treatment with melatonin. When administered orally, melatonin is mainly absorbed in the rectum and the ileum; it has a half-life of about 0.45–1 h and is extensively inactivated in the liver by phase 2 enzymes. Melatonin (MEL) concentration varies from picograms to ng/mL in fermented beverages such as wine and beer, depending on the fermentation process. These low quantities, within a dietary intake, are enough to reach significant plasma concentrations of melatonin, and are thus able to exert beneficial effects. Melatonin has demonstrated antioxidant, anticarcinogenic, immunomodulatory and neuroprotective actions. These benefits are related to its free radical scavenging properties as well and the direct interaction with melatonin receptors, which are involved in complex intracellular signaling pathways, including inhibition of angiogenesis and cell proliferation, among others. In the present review, the current evidence on the effects of melatonin on different pathophysiological conditions is also discussed.

## 1. Introduction

Melatonin (MEL) is a neurohormone (N-acetyl-5-methoxytyramine) from the pineal gland that is produced as secondary metabolite in the plant kingdom. Moreover, MEL synthesis occurs from tryptophan, 5-hydroxytryptophan, serotonin, and ultimately N-acetylserotonin. In addition, MEL can similarly be produced by O-serotonin methylation followed by N-methoxytryptamine acetylation in yeast [1,2].

MEL has been described in seeds such as rice and sweet corn, roots, leaves and fruits of a considerable variety of plants. In fact, it is present in some fruits [3]. The presence of MEL has also been described in olive oil, especially in extra virgin olive oil, and in sunflower oil [4]. In addition, the presence of MEL in grapes and wines has been recently described. Iriti et al. [5] detected various amounts of MEL in different grape varieties. These authors described concentrations from 0.005 ng/g to 0.9 ng/g. Moreover, MEL has been found in even higher concentrations (245–423 ng/mL), in ten single-varietal wines [6]. MEL and its isomers, despite not being present in grape musts, were detected in different finished wines. Finally, some experimental winemaking methods revealed that MEL is formed after inoculation with yeast, with the role of Saccharomyces cerevisiae being crucial [6].

MEL can even exert several beneficial effects for health in humans, demonstrating antioxidant, anticarcinogenic, immunomodulatory and neuroprotective action [3]. On the other hand, the biological activities of the most important MEL metabolites, N-1-acetyl-N-2-formyl-5-methoxyquinuramine (AFMK) and N-1-acetyl-5-methoxyquinuramine (AMK), have also been studied. AFMK is considered a powerful antioxidant, providing protection to DNA and lipids against oxidative damage through diverse metabolic pathways. Instead, AMK is also a potent antioxidant that is able to inhibit the biosynthesis of prostaglandins by binding to diazepam receptors [7].

As is the case with many secondary metabolites, MEL is able to stimulate endogenous antioxidant enzymes and/or counteract free radicals (both in vitro and in vivo) [1]. Moreover, MEL is capable of capturing reactive oxygen species, such as peroxynitrite [8] and even hydrogen peroxide in a dose-dependent way [9], as well as demonstrating antioxidant capacity in vitro through the ABTS^+^ method [10].

Studies in vivo have also demonstrated its antioxidant effect. When administered in mice it was observed that MEL was able to reduce chronic oxidative stress related to aging [11], and that it could even reduce blood pressure in men with chronic hypertension [12].

The role of MEL as a neuroprotective agent is also relevant. It has been successfully tested in sleep disorders, helping to restore circadian rhythm, and is especially effective in patients with neurodegenerative diseases. Several trials have been conducted with the aim of mitigating the consequences of diseases such as Alzheimer’s, Parkinson’s, Huntington’s disease or amyotrophic lateral sclerosis, obtaining satisfactory results [3]. Finally, MEL has also managed to inhibit the fibrinogenesis process significantly [13,14].

The amphipathic character of MEL allows it to cross physiological barriers, being present in the cytosol, mitochondria and different biological membranes [15]. Therefore, MEL provides the antioxidant defense where it is needed.

In order to establish the therapeutic use of melatonin for possible improvements in health, it is crucial to establish its bioavailability in humans. Secondly, considering the dietary sources of melatonin, it is desirable to evaluate precisely its content in food products and the expected intake. Finally, its positive effects must be evaluated, preferably in human randomized clinical trials, which are the best to establish cause–effect relationships. Therefore, with this strategy in mind, the present review aims to link these three areas, focusing on wine and beer as food sources of melatonin. The review evaluates the bioavailability of melatonin and resulting metabolites, the presence of melatonin in wine and beer, products that include a high range of bioactive phytochemicals and finally the different benefits related to the treatment with melatonin, studied both in animal models as well as mainly in humans.

## 2. Melatonin, Bioavailability, and Pharmacokinetics

### 2.1. Bioavailability

MEL absorption shows site dependency in the intestinal tract, being mainly absorbed in the rectum and the ileum. However, the absorption behaviors of MEL are more complicated when simultaneously dosed with excipients. Moreover, the absorption rate of MEL can be affected by the tissue damage, the formation of micellar complexes—characterized by NMR analysis—, the distribution of the particle size. For example, higher sodium cholate and sodium oleate concentrations are able to decrease the absorption of MEL due to the formation of micellar complexes, notwithstanding histological tissue injury. It is important to note that new insights in absorption of MEL and effects of common pharmaceutical excipients could be valuable for oral dosing in clinical pharmacokinetics in addition to stablish new oral formulations.

The elimination stage of orally administered MEL is biphasic, with a half-life of about 0.45–1 h depending on the administered dose, a fact that could be probably explained by the possible saturation of catabolic pathways. The differences observed on elimination half-life regarding age are probably due to the differences in metabolism. Finally, MEL is partially insoluble in water and it could influence the dissolution into the gastrointestinal tract [16].

MEL is inactivated in the liver by 6-hydroxylation, followed by sulfate and glucuronide conjugation, which leads to its main urinary metabolite 6-sulfatoxymelatonin [17]. It is important to note that liver clears more than 90% of the plasmatic MEL and is the primary site for its metabolism. MEL is originally hydroxylated before being excreted in urine as sulfate and, to a minor extent, as glucuronide conjugates.

The excretion of urine 6-sulfatoxymelatonin meticulously matches plasmatic MEL profile and only about 1% of circulating MEL is finally excreted in the urine without changes. 3-Hydroxymelatonin is also detected in urine and could denote the generation of the OH^•^ radical. In addition to reduced pineal secretory activity, subjects with liver cirrhosis show a minor MEL clearance, with the consequent postponed rise of plasmatic MEL peak and augmented daytime concentration of MEL.

The plasmatic profile of MEL shows wide intersubject variation. However, it is actually reproducible daily in the same person on behalf of one of the most robust circadian rhythms. It provides a good evaluation of MEL secretion if absence of renal or hepatic function is normal.

In some people, the nocturnal secretion of MEL is really short or even absent. The concerns of reduced MEL secretion on susceptibility to rhythmic organization and morbidity are still unknown. Plasmatic MEL is mainly bound to albumin (70%) and, to a minor extent, to orosomucoid, reaching every tissue—and is able to cross the blood–brain barrier—and modulating brain activity [18].

Matthews et al. [19] studied MEL metabolism in healthy volunteers. Urinary concentration of 6-sulfatoxymelatonin was similar in men and women (ranging from 6.3 to 30.9 mg/24 h). There was marked rise in nocturnal 6-sulfatoxymelatonin with > 80% of the total 24 h excretion comprised in the first urine sample. Following intravenous administration of MEL (1 mg), 99.7% was excreted within the first 24 h.

### 2.2. Pharmacokinetics

The bioavailability of MEL has been studied and very well described by the literature, showing an absorption reaching up to 33% after oral administration. However, the efficacy of MEL is still uncertain, needing more studies that to prove the good availability of MEL.

MEL has a noticeable hepatic uptake, reducing bioavailability if it is orally administered. Moreover, MEL was determined for healthy individuals and patients with cirrhosis, establishing a normal MEL production of 28.8 mg/day for the healthy population, and 12.3 mg/day for cirrhotic patients [20]. Kennaway et al. [21] reported the effect of possible structural modifications of MEL on plasmatic MEL half-life, showing a major uptake of 6-hydroxyMEL sulfate [22]. Golovanov et al. [23] studied the pharmacokinetics of MEL administered orally, intravenously or intramuscularly in rabbits and dogs. The maximum plasmatic concentration was achieved earlier and clearance from the blood was faster in rabbits than dogs for both oral and intravenous MEL. In fact, dogs showed higher area-under-the curve after oral MEL administration than rabbits. Therefore, the bioavailability was bigger for dogs than rabbits. After intramuscular MEL treatment, bioavailability between the species showed to be similar in both cases.

The clearance of MEL has been studied in man after intravenous injection of 5 or 10 mg and after 5 h infusion of 20 mg, showing biexponential decline [24]. Le Bars et al. [25] reported plasmatic pharmacokinetics of MEL, showing maximum activity in the brain after the injection of 9.5 mg/kg. The results of that study confirmed that MEL freely crosses the blood–brain barrier and that 6-sulfatoxymelatonin is the major plasma metabolite that can be found. Cavallo et al. [26] performed a pilot study on adult males showing dose linearity, absence of saturation kinetics, and unaltered metabolism and urinary excretion for doses of 0.1, 0.5 and 5.0 mg/kg. The results of the pharmacokinetic study confirmed no significant gender differences in adults.

The review of the literature specifies that oral bioavailability of MEL in humans and animal models reveals that the apparent elimination half-life of MEL following intravenous dose of 3 mg/kg was 19.8, 18.6, and 34.2 min, respectively, in rats, dogs, and monkeys [27]. Fluvoxamine is a selective serotonin reuptake inhibitor that is known to increase plasmatic MEL concentration. However, it is not clear whether these effects might be attributed to increased MEL production or decreased removal of plasmatic MEL. The last hypothesis was examined by Hartter et al. [28], who coadministrated fluvoxamine to a 17-fold high serum concentration. Haertter et al. [29] studied the influence of concomitant caffeine intake on the pharmacokinetics of oral MEL, showing a noticeable effect of caffeine on the bioavailability of oral MEL.

MEL shows volatile absorption from the gastrointestinal tract and—as commented before—extensive first-pass hepatic metabolism. Therefore, oral bioavailability of MEL varies widely between different subjects. MEL distribution follows on open one—or two—compartments, while its elimination may be described by biphasic first order kinetics [23].

MEL is typically administered orally at doses of 1–5 mg, which leads to pharmacological concentration in plasma. It has been reported that oral administration of 0.3 mg given 2–4 h before bedtime leads to normal nighttime plasmatic MEL concentration [30]. In addition, 180 min after oral administration of 80 mg of MEL, plasma MEL concentration increases with an absorption half-life of 0.4 h and an elimination half-life of 0.8 h [31]. There have been observed huge interindividual variations among subjects for MEL absorption (reaching even 25-fold differences). It is important to note that plasmatic MEL and half-life change depending on the dose, time of administration, and the type of oral preparation used [32]. Moreover, the MEL receptor sensitivity is increased between 17.00 h and 20.00 h [33]. The preservation of the high-amplitude circadian rhythm of MEL, with its high nocturnal concentration and low daytime concentration, is critical for the therapeutic treatment with exogenous MEL [30,31,32,33].

## 3. Melatonin in Fermented Products

MEL concentration varies from picograms to ng/mL of product in fermented beverages such as wine and beer [6,27,28]. Although the concentration is low it seems that these concentrations are sufficient in the dietary intake to measure their effects by different methods [29]. Although the MEL content can vary in different non-fermented products, it seems clear that the alcoholic fermentation process is decisive for the formation of MEL, since it is generated after inoculation with yeasts, the role of Saccharomyces cerevisiae being crucial [6,34].

### 3.1. Quantity of MEL in Wines

MEL concentration varies with fermentation, presenting its highest value between the first and second day of fermentation [23,30,31]. It should be taken into account that several factors can affect the concentration of MEL in red wine, such as agrochemicals used, winemaking practices, fermenting microorganisms or even the composition of the grapes used [30,32,33,35].

Several authors have described the presence of MEL in wines, as shown in Table 1 [30,32,33,36]. Mercolini et al. found values of 0.4 and 0.5 ng/mL in Sangiovese red wines and Trebbiano white wine [37], and found 0.3 and 0.5 ng/mL in varieties of Albana grappa and grape juice [38]. Stege et al. found values of 0.16 ng/mL for Malbec red wine, 0.24 ng/mL for Cabernet Sauvignon red wine and 0.32 ng/mL for Chardonnay white wine [39]. Vitalini et al. [40] values of 4.1 and 8.1 ng/mL for Gropello and Merlot wine varieties, respectively. Rodriguez-Naranjo et al. found values between 74 and 322 ng/mL for Presses wines (Sauvignon, Merlot, Syrah, Tempranillo and Tintillo de Rota) and between 250 and 340 ng/mL for Racked wines (Sauvignon, Merlot, Syrah, Tempranillo, and Tintilla de Rota) [6]. Vitalini et al. found values between 0.14 and 0.62 ng/mL for varieties of single-variety red wines, 0.05–0.31 ng/mL for polyvarietal red wines, 0.18 ng/mL for white wine and between 0 and 0.31 ng/mL for Dessert wines and 0.11–0.13 ng/mL for Modena balsamic vinegars [35].

In a study where tryptophan and certain metabolites, including MEL, were simultaneously analyzed in several types or red wine, MEL values ranged from 0.038 ± 0.001 g/L to 0.063 ± 0.004 g/L [35], data consistent with those shown by Vitalini et al. as 0.05–0.062 g/L [35].

It should be noted that the presence of MEL in the grape is not always reflected later in the wine, as shown in a study of Gómez et al. [43] where the concentration of MEL of the grape was 120–160 ng/g and yet in the wine of those grapes there was no longer MEL but a MEL isomer that ranged from 18 to 24 ng/g.

It is important to remark that the oral bioavailability of MEL after intake of a glass of wine is not known, which is not the case for polyphenols, perhaps due to the complex food matrix that may influence the absorption of active metabolites [40], since it is in the form of a supplement that is consumed at high doses and has been known for years [37,44]. However, the presence of ethanol seems to improve the amount of MEL, given its solvent ability, by improving the permeability of membranes [42].

Varoni et al. evaluated the serum MEL levels after administering a wine enriched with MEL vs a wine with placebo in humans and it was observed that the maximum MEL concentrations were within 60 min, being 8.7 ± 2.2 pg/ min for the MEL group and 6.7 ± 0.6 pg/ min for placebo wine, obtaining an area under the curve of 993 ± 162 vs. 745 ± 88 pg/min of the MEL vs. placebo group, respectively, without observing significant differences. As for salivary concentration, the peak was reached at 45 min after MEL intake, also without statistical significance, returning after 120 min to placebo levels [40].

### 3.2. Quantity of Melatonin in Beer

Beer is part of the usual consumption of a large number of people, and is characterized by having a wide variety of bioactive nutraceutical and phytochemical compounds such as polyphenols and antioxidants [41] presenting B-complex vitamins, ascorbic acid, citric acid, etc. As for the MEL content present in beer, a study of 18 brands of beer present on the market, featuring different alcohol content, showed how all beers featured MEL being directly proportional to the alcohol content at higher MEL content, with values varying from 51.8 ± 2.2 pg/mL—non-alcoholic beer—to 169.7 ± 8.7 pg/mL—beer [43]. Such an effect could be due to the solubility of MEL in alcohol. Another study of 20 varieties of beer showed that MEL content ranged from 58 ± 1.44 pg/mL in beverages with 0–2% volume of alcohol to 169 ± 2.4 pg/mL in beers with 7–7.8% volume of alcohol, data consistent with the previous study. In addition, another study produced beer by hand and measured the concentration in the different manufacturing processes obtaining a final value of 333 pg/mL in a beer of 5% volume of alcohol after the second fermentation, which are values that are three times higher than commercial beers [45]. In terms of composition, high levels of MEL (339 ± 9 pg/mL) were found in barley of concentrated musts versus low amounts in hops 33 ± 10 pg/mL [45]. It seems that what happens in wine, where the concentration of MEL is attributable to the fermentation process rather than the content of the grapes, is different in beer, where it can be attributed to the content in barley.

## 4. Melatonin and Antioxidant Activity

The action of MEL in the direct uptake of free radicals has been recognized for decades [46]. A metabolite of MEL, cyclic-3-hydroxymelatonin (c3OHM), produced when MEL is able to remove two free radicals, has also been identified [47]. Since this discovery, the direct sweeping actions of free radicals through MEL and its metabolites have been described [48,49,50]. MEL stimulates the antioxidant enzymes of the organism such as glutathione peroxidase, and glutathione reductase [51,52] regulates the increase in glutathione [53], improving the reducing power of tissues and fluids [54], neutralizing the nitrogenous toxins such as nitric oxide, and peroxynitric anion, responsible for nitrosative damage [55,56], is capable of chelating heavy metals [57] and suppresses the nitric oxide synthase, known as prooxidative enzyme [58,59]. A positive correlation was found in humans comparing night and day MEL concentrations and antioxidant capacity of blood even at physiological doses [60].

MEL, c3OHM and the rest of their metabolites are excellent scavengers of free radicals [61] which is why it has been called the antioxidant coat of MEL [23], which is capable of neutralizing up to 10 types of free radicals, unlike the classic captors that have the ability to capture only one [62].

Regarding the chelation of metals, Limson et al. showed that MEL binds to zinc, lead, copper, iron, aluminum and cadmium ion a similar way to metallothionine, the interaction with these metals being dependent on the concentration of MEL [57]. MEL chelates both Fe^3+^ and Fe^2+^, preventing the formation of the hydroxyl radical. MEL and its metabolites are capable of chelating Cu^2+^, completely inhibiting oxidative stress, preventing the first step in the Haber–Weiss reaction, neutralizing the formation of the highly oxidizing hydrozyl radical [57,61].

MEL and its metabolites, in addition to neutralizing a large number of reactive molecules, modulates the activity of certain enzymes that neutralize reactive oxygen and nitrogen species produced, as summarized in Table 2. Limiting the leakage of electrons from the mitochondrial respiratory chain, thus reducing the amount of oxygen molecules to superoxide anion [63], due to its anti-inflammatory character, given that inflammation generates free radicals, MEL reduces the oxidative damage [64], and thanks to the regulation of circadian rhythms, oxidative processes with lower production of oxidant molecules are regulated [65].

Mitochondria are one of the main sites for the production of reactive oxygen species [66] which must be detoxified before they damage these organelles. Due to the high production or reactive oxygen species in the organelles, a key step in preventing oxidation would be to get antioxidants to target and attach to the mitochondria [67]. Lowers et al. performed a comparison of MEL against two antioxidants directed to mitochondria (MitoQ and MitoE) in mice after an aggressive prooxidant treatment, in order to reduce inflammation and oxidative damage, observing that MEL was the most effective measure, so MEL was qualified as an endogenous antioxidant directed to mitochondria [68].

It seems that MEL, which is ubiquitously distributed throughout the organism, exerts action in each and every one of the organs and tissues of the organism, and due to its pleotropic character it is really effective in combating oxidation through a highly coupled antioxidant defense axis [68].

## 5. MEL and Prevention of Neurodegenerative Diseases

Neurodegenerative diseases are characterized by the progressive decline of brain structures and normal function. The degeneration of some selective neuron regions increases the progression to notably cognitive symptoms in Alzheimer’s disease (AD), frontotemporal dementia or even primarily motor signs in Parkinson’s disease (PD), amyotrophic lateral sclerosis (ALM), or Huntington’s disease (HD) [69].

The effect of MEL on mitochondria plays a key role in its neuroprotective function in neurodegenerative processes. MEL plays an essential role in reducing the processes that cause neuronal death such as chronic inflammation, disturbance of the circadian rhythm, increment of oxidative stress, autophagic deficiency and mitochondrial damage with loss of ATP production capacity and neuronal death. Diverse experimental models of AD, PD and HD denote the efficacy of MEL in antagonizing the disease evolution and/or the mitigation of some symptoms. In fact, MEL secretion has been found to be altered in AD and PD.

It is currently known that oxidative stress is a major factor favoring the development of neurodegenerative diseases, and free radicals play a central role in the physiopathology of these diseases, as with all neurodegenerative diseases [70]. MEL has shown neuroprotective capacity in neurodegenerative disorders with few side effects, even at high doses [71].

### 5.1. Alzheimer’s Disease and Melatonin

AD is characterized from a physio-pathological point of view as extracellular accumulation of β-amyloid peptide (Aβ) in amyloid plaques and neurofilament tangles, formed by fibrillar aggregates of hyperphosphorylated tau proteins. The accumulation of beta amyloid is produced by an imbalance between Aβ clearance and production. Slow wave sleep is related to the formation of beta amyloid and tau because they influence clearance mechanisms. The mechanisms causing these neuropathological modifications are still uncertain, but are possibly produced by underlying both environmental and genetic factors. The main described symptoms of AD are related to memory loss—mainly associated with language insufficiency—, personality disorders, and alterations in the sensory–motor association functions [72].

Aβ is produced via amyloidogenic managing by β- and γ-secretase. MEL is able to inhibit Aβ production and aggregation, as reported in in vivo and in vitro studies [73]. In Aβ-induced animal models, MEL was able to reduce the production of Aβ and reduces apoptosis by the decrease in caspase-3 activity and the increment of B cell lymphoma-2 (Bcl-2) expression in the brain. Furthermore, the pre-treatment with MEL prevents Aβ-induced increase in the levels of bax mRNA, enhancing the expression of bcl-2 [74].

The previously mentioned hyperphosphorylation of tau protein disturbs the attachment of tau to the microtubules, thus disturbing the stability of the microtubules. MEL is able to considerably enhance the hyperphosphorylation of tau induced by the calyculin A (CA), wortmannin and the okadaic acid in diverse neuronal cells. Moreover, MEL attenuates the hyperphosphorylation of tau by the regulation of proline-directed serine/threonine kinases, for example GSK-3β and cyclin-dependent kinase 5 (CDK5); non-proline-directed serine/threonine kinases—including PKC, PKA and death-associated protein kinase 1 (DAPK1)—and protein phosphatase PP-2A. Furthermore, MEL reduces oxidative stress and attenuates the hyperphosphorylation of tau by the inactivation of GSK-3β. Although the source of the neurodegeneration in AD is still unclear, the three major factors leading to AD are free radical damage, mitochondrial dysfunction, and excitotoxicity [75].

This a bidirectional relationship between neurodegenerative diseases and sleep, which is a key contributor to the neuropathology. In fact, the sleep deficiency for only one night or the interruption of non-REM sleep is related to increased levels of Aβ1–42 and Aβ1–40 in cerebrospinal fluid (CSF) [76,77].

The decline in MEL production observed in the elderly population may contribute to age-related neurodegenerative diseases, and it is suggested as one of the main motives for the progress of AD. A decline in the concentration of MEL in the CSF matches the progression of AD, making the brain cells more susceptible to oxidative injury [78].

MEL can act as a potent antioxidant scavenger of OH radicals and other radical oxygen or nitrogen species (ROS and RNS), and that gives rise to the cascade of metabolites mentioned above that share antioxidant properties. MEL can also stimulate gene expression of different antioxidant enzymes and inhibit the production of prooxidant enzymes. Particularly, MEL enhances glutathione peroxidase (GPx) and glutathione reductase (GRd). MEL contributes to the maintenance of normal brain glutathione via γ-glutamylcysteine synthase and glucose-6-phosphate dehydrogenase. MEL has shown even better capacity that vitamin C and E in protection against oxidative injury and neutralizing free radicals [79,80]. All together, these factors make MEL a potentially useful compound in the management of neurological disorders related to oxidant damage.

Subramian et al. [81] reported the effect of a 45 days length administration of MEL at two different doses (0.5 and 1.0 mg/kg) on lipid peroxidation, antioxidant status and lipid profile in the brain and liver in rodents. Both doses of MEL led to a significant reduction in lipid peroxidation. Moreover, the plasmatic levels of cholesterol, phospholipids, triglycerides and free fatty acids were reduced. The treatment with MEL also incremented antioxidant capacity of the brain and liver as well as increasing glutathione levels.

It is noteworthy that the inflammatory component in AD is evidently different compared to normal inflammation [75]. Numerous authors have reported that MEL encourages cognitive function and brain flexibility against neurodegenerative processes. Moreover, the administration of a daily dose of 10 mg/kg of MEL induces a reduction in NF-κB and proinflammatory cytokine expressions in healthy nontransgenic (NoTg) and AD transgenic (3xTg-AD) mice. Moreover, the SIRT1 pathway of longevity and neuroprotection was also triggered in both kinds of mice [75].

This different actions of MEL in the central nervous system make this indoleamine hormone a hopeful molecule against neurodegeneration and cognitive diseases [82].

### 5.2. Parkinson Disease and Melatonin

PD is a chronic and neurodegenerative pathology occurring with motor and nonmotor symptoms. There are many pathways that are related to PD, including autophagy, apoptosis, inflammation, oxidative stress, α-synuclein aggregation, inflammation, and neurotransmitters changes [83]. Alpha-synuclein have been identified in familial PD and constitutes one of the chief components of Lewy bodies in intermittent cases of PD. Moreover, oxidative stress, ROS and NOS from mitochondrial impairment and/or dopamine metabolism are considered determinant in PD etiopathogenesis [84].

Singhal et al. [85] showed how MEL is able to inhibit oxidative stress and apoptosis by the increment on the concentration of Dopamine and by preserving dopaminergic neurons in mice models of PD. MEL has been found to improve neurotoxicity by increasing the ubiquitination of α-synuclein due to the prevention of autophagy and α-synuclein aggregation [86]. Su et al. [87] reported that pretreatment with MEL reduced axon and dendritic length of neuron induced by MPTP, recovering the dopaminergic neuronal damage due to the inhibition of CDK5-mediated autophagy and SNCA aggregation.

Moreover, MEL is able to increase the treatment effects of L-DOPA, decreasing the dosage in animal model of PD, which represents a superlative adjuvant to L-DOPA treatments in PD. Furthermore, MEL was able to improve some nonmotor symptoms in PD patients. MEL plays a neuroprotective role in neurodegenerative diseases by the regulation of autophagy, representing a new emerging treatment opportunity [88].

Therefore, the administration of MEL derives from the inhibition of some pathways related to oxidative stress, autophagy, α-synuclein aggregation, apoptosis, inflammation, and dopamine loss in PD. In fact, preclinical studies showed that MEL may be a perfect and ideal coadjutant for the treatments with L-DOPA in PD; however, there is still a lack of evidence and more clinical studies seem to be needed [83].

### 5.3. Melatonin and Amyotrophic Lateral Sclerosis

ALS is a deadly, progressive neurodegenerative disease that is characterized by the loss of motoneuron function in the brainstem, brain, and spinal cord. Many common pathogenic mechanisms can be found between ALS and the ageing, such as protein aggregation, oxidative stress increment, metabolic deficiencies, decline of mitochondrial and microglial function, and general inflammation [89]. The cell death mediated by caspase contributes to the pathogenesis of the motor neuron degeneration (in mutant SOD1G93A mice) of ALS, accompanied by other factors such as general inflammation and oxidative injury. Zhang et al. [49] showed that MEL can exert a neuroprotective capacity in ALS, due to its faculty to inhibit the caspase-1/cytochrome c/caspase3 pathway and to rescue MT1 expression. Weishaupt et al. [71] reported that MEL is able to reduce the cell death in cultured motor neurons induced by glutamate.

### 5.4. Melatonin and Huntington’s Disease

MEL can be useful in HD patients due to its antioxidant capacity, which exert neuro-protective effect, and antiapoptotic properties. One study using a mouse genetic model showed the neuroprotective capacity of MEL, leading to increased life expectancy of these animals. It was also observed that MEL MT1 receptors are progressively deteriorate over the course of the disease, leading to gradual depletion of MT1 receptors. This depletion led to increased vulnerability to cell death. However, it the exogenous administration of MEL neutralizes this depletion, it may consequently be used to improve the prognosis of the disease, but not to cure it [90].

### 5.5. Melatonin and Multiple Sclerosis

MS is an autoimmune progressive neurodegenerative illness prompted by a response against myelin. MS represents the most predominant demyelinating disease of the Central Nervous System (CNS) in young adults. MS is characterized by a chronic general inflam-mation and Blood–Brain Barrier disruption. Moreover, MS leads to lymphocyte infiltration into the CNS, which causes myelin sheath damage, besides gliosis, and axonal loss. MS patients frequently suffer fatigue, mood disorders, cognitive deficiency and sleep disorders [91]. The neuroprotective effects of MEL are mainly due to its antioxidant capacity; how-ever, some recent evidence proposes that activation of the MEL receptors (MT1 and MT2) may also play a determinant role [61]. Recent evidence suggest that MEL secretion can be dysregulated in MS patients, proposing that MEL could be a potential target for therapeu-tic intervention in the patients. Moreover, MEL protects against neurodegeneration and senescence associated with oxidative stress through the reduction in p-p38, inhibition of p16INK4α and increase in SIRT1 [92].

Long et al. [91] reported that the treatment with MEL significantly reduced demye-lination of axons, besides showing minor loss of mature oligodendrocytes and axonal damage. Moreover, it can activate the Nrf2/ARE pathway leading to the increment of an-ti-oxidant enzymes HO-1 and NQO1 expression. MEL and baclofen concomitant treat-ment is able to increase the antiinflammatory cytokine IL-4, reduce IFNγ serum levels, in addition to inducing remyelination in the pMOG-induced EAE model [93]. Finally, the administration of 5 mg of MEL during 90 days in MS patients was able to reduce serum total oxidative status and increase total antioxidant capacity through the induction of the activity of glutathione peroxidase and SOD [94]. Therefore, MEL may be a promising mol-ecule for the treatment for MS or other autoimmune diseases in the near future.

## 6. Melatonin and Cancer

MEL may act as protective molecule against cancer. Firstly, MEL can act by scavenging capacity and preventing damage to nuclear DNA. If the damage produced is large and it is not repaired, the damage accumulated along the whole constitutes the main cancer cause in the elderly [95]. MEL has proved to be useful in the treatment of cancer, as have other bioactive molecules with scavenging capacity. Furthermore, MEL can exert its anticancer effects in the first stages of cancer, alleviating the side effects due to its a chronobiotic effects, and improves the wellbeing of the radio and chemotherapy [96].

One the main targets of MEL is the prostate, leading to minor occurrence of prostate cancer cells (PCa) proliferation, stimulating neuroendocrine differentiation. The treatment with MEL at the first stage of cancer was able to prolong the survival of TRAMP mice by 33% [97].

There is seems to be a link between Sirt1 and circadian rhythms, as reported by Jung-Hynes et al. [95]. They showed that the disruption of the production of MEL by the pineal gland deregulates the circadian rhythm machinery and increases cancer risk. These authors reported that MEL was able to significantly inhibit Sirt1 protein, leading to a significant reduction in the proliferative potential of PCa cells, but not of normal cells.

Furthermore, the antiestrogenic capacity of MEL may enhance its ability to reduce the proliferation of some types of hormone-related cancers, such as breast cancer [98]. MEL is effective in the reduction in endothelin-1 (ET-1) concentration in the brain of patients suffering from a stroke. ET-1 is considered an important molecule for the promotion of angiogenesis and has been related to the control of cancer expansion [99]. MEL has antiproliferative capacity, which has been validated in both ERα-positive and ERα-negative human breast cancer cell lines. In fact, Santoro et al. [100] showed that MT1 and MT2 receptors of MEL are required to prevent the damage in p53-mediated DNA. Finally, MEL has the ability to induce phosphorylation of p53 (increased in breast cells) leading to the inhibition of cancer proliferation and the prevention of DNA damage accumulation [101].

The main strategy of cancer cells to avoid apoptosis is the overexpression of apoptosis-resistant molecules. The treatment of pancreatic cancer cells with MEL leads to apoptosis mediated by down-regulation of Bcl-2 and up-regulation of Bax. In fact, human myeloid leukemia cells that were treated with MEL inhibit the progression of cancer by Bax up-regulation and Bcl-2 down-regulation [102,103]. The MEL capacity to induce apoptosis encompasses other types of cancer, such as human hepatoma, murine gastric cancer and prostate cancer [101]. The mammalian target of rapamycin (mTOR) is a protein kinase involved in the control of some determinant physiological pathways related to cancer processes, such as cell growth, proliferation, protein synthesis, metabolism, and autophagy. The treatment with MEL of tumor bearing rats during 60 days led to a decrease in tumor size that was related to reduced levels of mTOR [104].

Moreover, angiogenesis is critical and determinant to tumor growth and provides dividing cells with the oxygen and nutrients needed in order to maintain cell division. Interestingly, the treatment with MEL led to the expression of VEGFR-2 and reduced micro-vessels concentration in mice [105]. Lissoni et al. [106] reported that orally administered MEL was able to reduce serum VEGF-2 levels in cancer metastasis patients. The angiogenesis suppression seems to be facilitated by the reduction in endothelin-1 [107]. MEL suppresses the estrogen receptor alpha (ERα)-positive via the MT1 receptor. MEL also has anti-metastatic actions concerning multiple pathways and inhibits p38 MAPK [108].

Therefore, there is abundant evidence supporting the multiple tumor-suppressing capacity of MEL, exhibiting a potent protective effect against chemotherapy, particularly through anti-gonadotropin and anti-estrogenic capacity. Owing to a low toxicity—even at high doses—and the huge variety of beneficial effects showing, MEL is a potential bioactive compound to be considered as complementary therapy for the treatment of different types of cancer [109]. The relevance of MEL in reducing the incidence of cancer relies on its radioprotector capacity, as well as its possible use under the situation of prolonged low dose radiation exposure, which could rise the risk of developing cancer [110].

## 7. Melatonin and Circadian Rhythm

Circadian rhythms are biological cycles in the organism that continue in a period of approximately 24 h and are generated by the neural centers in the suprachiasmatic nucleus (SCN) of the hypothalamus. Circadian rhythms are synchronized to light/dark as external stimuli (zeitgeber). The most prominent circadian rhythm in humans is the sleep/wake cycle, as well as the biological timing of release of certain circulating hormones, such as MEL.

Different tissues and organs synthesize MEL for local use, but, as a hormone, it is secreted mainly by the pineal gland and released to blood and cerebrospinal fluid. This pineal production is precisely controlled by the neural centers in the suprachiasmatic nucleus (SCN) of the hypothalamus [111]. In this way, MEL is a hormone with clear fluctuations across the day and the night, tightly synchronized to the light/dark cycle.

MEL production is circumscribed to the night, irrespective of the level of activity or rest. In fact, MEL has been defined as the “chemical expression of darkness” [112], and light during the night blocks its production. During the light period, the retina receives the light information that is transferred by the optic nerve to the SCN and inhibits it. At dim light or in the darkness, the information sent to the SCN is translated in a release of noradrenaline through sympathetic nerves, which activate the pineal gland and cause MEL synthesis. MEL is then released to the blood vessels and general circulation.

MEL can be detected in plasma and saliva, and its major metabolite, 6-sulphatoxy-MEL, can be detected in urine. It is currently measured in saliva, due to the feasibility of sample extraction that allows frequent sampling, for monitoring circadian rhythm. A common parameter used as marker of circadian rhythm is the dim light MEL onset (DLMO). DLMO indicates the beginning of endogenous MEL production, about 2 h before bedtime, provided that the light is dim. At this moment, MEL concentration in plasma exceeds 10 pg/mL (3 pg/mL in saliva), whereas its daytime levels are in the range of 1 pg/mL. The 24 h-urine excretion profile of 6-sulphatoxy-MEL is also used to confirm circadian rhythm sleep–wake disorders [113]. In most people, plasma MEL levels increase to a reach a maximum peak at around 03.00 h am and decline to basal levels by around 07.00–08.00 h am [114].

Monitoring the circadian rhythm of MEL requires numerous samples to be taken at night, since during the day its levels are very low and current measurement techniques present difficulties in terms of sensitivity. Attempts are being made to validate and compare the methods to analyze this biomarker in order to improve their quality parameters [113,114].

After distribution in the body, MEL binds to membrane receptors (MT1 and MT2, also known as MTRN1A and MTRN1B, respectively), which belong to the family of G-protein-coupled receptors. MT membrane receptors are located in brain (mainly MT2) and other peripheral tissues. These receptors are mainly coupled to Gi proteins and their activation inhibits the adenylate cyclase pathway, reducing intracellular levels of cAMP and decreasing activation of protein kinase A. In parallel, MT1 coupled to Gq protein leads to phospholipase C (PLC) activation, increasing intracellular Ca^2+^. MT2 activation also inhibits cGMP levels [115]. Due to its lipophilic character, MEL can enter the cell and binds to retinoid nuclear receptors RZR/ROR, as well as to cytoplasmic proteins, such as calmodulin, protein-kinase C (PKC) and MT3. MT3 is a quinone reductase 2 enzyme, involved in the maintenance of redox-balanced status. It seems that MEL functions as a cofactor of this enzyme [116].

The observed effect varies depending on the target organ. In pancreatic islet cells, the interaction of MEL with MT1 and MT2 receptors decreases glucose-stimulated insulin release, via adenylate cyclase/cAMP inhibition. In addition, MT1 activation in β-cells induces phosphorylation of insulin receptor, which, via the PI3K and MAPK phosphorylation and activation, controls insulin synthesis and release [117]. In this way, MEL contributes to the circadian profile of insulin secretion, synchronized with the activity–feeding/rest–fasting states.

In adipocytes, the interaction of MEL with MT1 and MT2 receptors and the activation of phospholipase C pathway promotes the browning of white adipose tissue, thus increasing the size of the brown adipose tissue (BAT). BAT adipocytes contain high amounts of mitochondria, a high rate of β-oxidation and dissipate energy by heat. [118]. MEL enhances BAT metabolic activity and promotes thermogenesis [119]. Lipolysis and increased mitochondrial function was observed in mice adipocytes [120]. In humans, MEL replacement (3 mg/day) for 3 months in MEL-deficient patients increased BAT size and activity [121].

In insulin-sensitive cells, such as adipocytes, skeletal muscle and myocardial cells, MEL induces, via MT receptors, the phosphorylation of the insulin receptor and further intracellular substrates, what is translated in an increase in the expression of glucose transporter GLUT4 and a higher glucose uptake by these cells. The decrease in levels in MEL is followed by a minor GLUT-4 gene expression and hence a lesser uptake of glucose by muscle, adipocytes and myocardial cells, that can be reverted by MEL replacement therapy [111].

In this way, by the interaction with MT receptors in different body organs, MEL acts as a central synchronizer or “internal zeitgeber”, playing a major role in the regulation of the circadian biological timing of different physiological phenomena. MEL integrates the sleep/wake cycle with the energy metabolism, that follows a circadian pattern as well. In fact, the active phase of the day, with low circulating levels of MEL, is characterized by energy uptake by cells, use and storage. There is a high sensitivity to insulin and glucose uptake by tissues, glycogen synthesis and glycolysis and increased adipose tissue lipogenesis. During the rest phase, there is a fasting period in which energy from stores is used for the maintenance of physiological functions. This phase is characterized by insulin resistance, gluconeogenesis and glycogenolysis, adipose tissue lipolysis and leptin secretion [111]. Deficiency in MEL production leads to a disturbance of the light/dark cycles—chronodisruption—that has been related to increased risk of insulin resistance and type 2 diabetes.

## 8. Melatonin and Cardiovascular Health

### 8.1. Chronodisruption and Cardiovascular and Metabolic Disorders

Maintenance of a proper circadian sleep–wake cycle is fundamental for cell function, the neurological repair system and neural plasticity. Levels of different hormones, such as MEL, are governed by the sleep/wake cycle, as well as by an endogenous timing system and both systems are generally well coordinated. However, there are conditions in which the sleep/wake cycle and the endogenous timing system are uncoupled. The frenetic pace of life we lead today, with shift-work or long working hours and leisure activities in wide time slots, leads us to 24/7 schedules that can contribute to imbalance the sleep–wake circadian rhythm.

If activity/rest periods of the individual are not aligned with the light/dark cycles (chronodisruption), it can result in a misbalance in day/night levels of circadian hormones, such as MEL, with negative consequences [122]. In fact, circadian rhythm disruption has been associated with a wide range of pathologies, including cardiovascular and metabolic disorders such as diabetes [123].

Observational studies in large cohort studies, such as the Nurses’ Health Study, have related an increased risk for ischemic stroke and hypertension with shift work [124]. Increased incidence of diabetes and obesity has been associated as well [125,126]. The increased risk of cardiometabolic diseases has also been reported in social jet lag conditions [127]. Social jet lag refers to the difference between the sleep–wake schedule of working and non-working days [128]. A study performed in an Hispanic community in the US (HCHS/SOL) showed an increase in insulin resistance in individuals with later sleep–wake timing, not aligned with the biological timing [129]. Exposure to more than the usual hours of light has been associated with an increase in BMI [130].

The high light exposure decreases MEL production, what is reflected in a poorer metabolism control. Reduced serum MEL levels are associated with elevated incidence of cardiovascular diseases in humans [131], such as hypertension [132] and heart failure [133]. In fact, low levels of the metabolite 6-sulphamethoxy-MEL in urine have been independently and inversely associated with the risk of hypertension in young women followed for 8 years [134], and with cardiovascular risk conditions such as diabetes. In most cases, in stages associated with low levels of MEL, adequate MEL replacement therapy repairs the metabolic alterations.

### 8.2. Melatonin Supplementation and Cardiovascular Health

MEL supplements and different MEL receptor ligands are available in the market for the treatment of sleep disorders or jet lag conditions, aimed to reset the circadian clock [115]. The European Food Safety Authority (EFSA) has published a Scientific Opinion on a health claim related to MEL and sleep disorders, considering that “MEL helps to reduce the time to fall asleep and the reduction of sleep onset latency might be a beneficial physiological effect. In order to obtain the claimed effect, 1 mg of MEL should be consumed close to bedtime” [135].

On the other hand, in vivo studies in animal models as well as human clinical trials have shown that MEL may help to reverse/ameliorate certain cardiovascular events. MEL has been supplemented in patients undergoing cardiac surgeries, with contrasting results. The infusion of 50 mg MEL in patients attending important vascular surgery, followed by oral administration of 10 mg in the first 3 nights after surgery, protected against reperfusion injury [136]. Proinflammatory and apoptotic markers in patients of coronary artery bypass surgery decreased with the MEL supplementation (10–20 mg/day), in part due to MEL ability to limit free radical damage [137]. On the other hand, there was a lack of cardiac protection with the intravenous and intracardiac administration of MEL [138,139].

Clinical trials have demonstrated that MEL supplementation at night (2.5 mg/day, 3 weeks), [12], (3 mg/day, 4 weeks), [140] reduces blood pressure in hypertensive patients. Similar results were observed on diminution of blood pressure in patients with metabolic syndrome, with doses of 5 mg/day for two months, who also showed a decrease in LDL-cholesterol and improvements in antioxidant status [132].

Some trials have failed to observe decreases in blood pressure with MEL supplementation, and authors explain this to be a result of administration during daytime. The long-term regular intake before sleep better reflects the normal rhythm MEL concentrations and may better restore the circadian rhythm of blood pressure. A meta-analysis of randomized controlled trials on nocturnal blood pressure showed that controlled-release MEL was effective in reducing blood pressure, in contrast to fast-release MEL, which had no effects. Controlled-release MEL provided MEL concentrations in blood that matched better with the physiological profile [141].

In fact, there are concerns about the mistiming of circadian based treatments like light and MEL, as they may worsen circadian disturbance and exacerbate other symptoms.

The cardioprotective effects of MEL have been attributed to varied mechanisms, including antioxidant properties, as well as the anti-inflammatory, immunomodulatory and direct vascular actions. It decreases platelet adhesion and the expression of vascular adhesion molecules, such as VCAM-1, ICAM-1 and endothelin-1 [142]. In cardiomyocytes, MEL activates different signaling pathways, including JAK2/STAT 3, PI3/Akt and Nr2, reducing cell apoptosis, inflammation and cell oxidation and thus preventing myocardial infarction injury [143,144]. MEL induces relaxation of the smooth muscle cells in blood vessels. The activation of MT2 receptors on endothelial cells involves activation of the phospholipase C pathway and the increase in cytosolic Ca^2+^ in endothelial cells, which further stimulates NO production and vasodilatation. Besides, MEL may have a central action, reducing the sympathetic tone in SCN (α1-adrenergic receptors) [145,146].

Anti-inflammatory properties of MEL have also been demonstrated, which can contribute to its positive effects on vascular function. In a clinical trial performed with obese women, the intake of a daily dose of 6 mg MEL for 40 days significantly decreased levels of pro-inflammatory cytokines TNF-α, IL-6, and c-reactive protein [147], compared to placebo. It seems that MEL affects different signaling pathways involved in the inflammation process, such as the activation of SIRT1, a secondary signal molecule that mediates anti-inflammatory actions [148,149].

**Figure 1:**
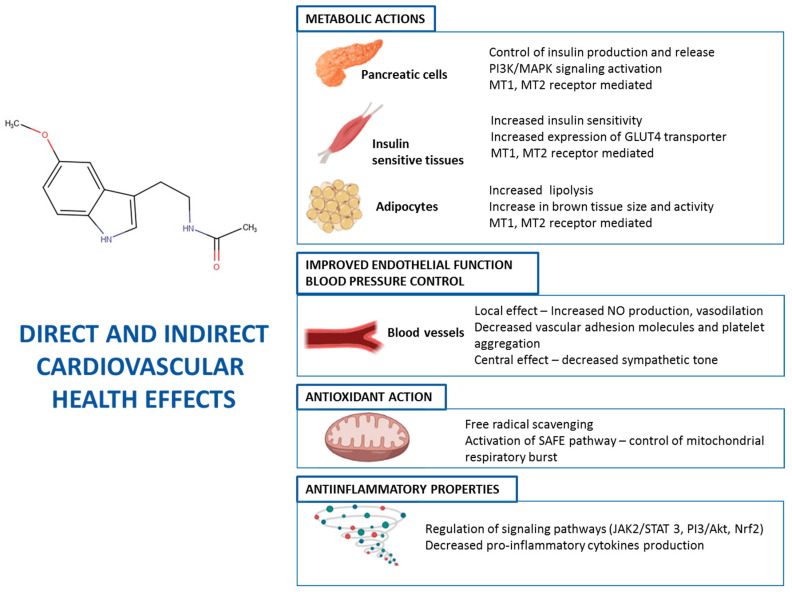
Mechanisms of action on MEL in different cells and tissues contributing to cardiovascular health.

Indirectly, due to its effects on energy metabolism via MT1 and MT2 receptors (see above), MEL helps to maintain the endothelial structure and function in blood vessels, by normalizing blood glucose and lipid profile. The prevention of formation of atheromatous plaques contributes to reducing the risk of ischemia in organs and cardiovascular pathologies. The glycemic control ameliorates the cardiovascular risk conditions, such as type 2 diabetes. Besides, MEL improves lipid serum profile, with increases in HDL-cholesterol, whilst LDL-cholesterol and triglycerides are reduced [150], thus reducing these cardiovascular risk factors. Figure 1 summarizes the main mechanisms of action of MEL on cardiovascular function.

### 8.3. Consumption of Melatonin-Rich Foods and Cardiovascular Health

Numerous epidemiological studies have shown that the Mediterranean diet provides benefits to cardiovascular health. Part of these effects can be attributed to the high intake of MEL-rich foods, such as red wine, nuts, olives and fish [151]. In fact, observational studies have shown that diets rich in walnuts reduce the risk of cardiovascular diseases.

Moderate consumption of wine has been correlated with a decreased risk of cardiovascular diseases (CVD), mostly related to its content in polyphenolic compounds. Besides, the contribution of MEL must be taken into account, whose levels in grapes increase during alcoholic fermentation [6,152].

Nutritional supplements contain much higher levels of MEL than foods; however, it has been demonstrated that the intake of foods rich in MEL significantly increases the plasma levels of this hormone. The intake of 200 mL of grape juice daily for 5 days significantly increased urinary levels of the metabolite 6-sulphatoxyMEL in young and middle-aged adults [153]. Similarly, increases in serum MEL concentration were observed after consumption of banana, orange and pineapple juices [154].

Most studies on the effects of MEL on cardiac health against ischemia-reperfusion have been performed at high concentrations, ranging from 1 to 50 µM. In contrast, other authors have evaluated the protective actions against ischemia-reperfusion injury of MEL, using realistic concentrations in such foods as red wine [155] and MEL, at this physiological concentration, significantly reduced infarct size. The mechanism behind this involves the activation of the prosurvival surviving activator factor enhancement (SAFE) pathway. The SAFE pathway involves the activation of the cytokine TNF-α and its receptor and the activation of transcription factor STAT3, which regulates levels of reactive oxygen species in mitochondria and the electron chain transport activity [156].

The consumption of MEL-rich foods provides significant amounts of MEL; however, clinical trials are needed to ascertain the impact of their intake in MEL’s actions on the cardiovascular system and whether the dietary MEL is enough for cardioprotection, or higher doses are needed. The consequent information can be of great value for the design and development of functional foods.

## 9. Conclusions

The current literature highlights the good bioavailability of melatonin in humans, leading to two active metabolites that should serve to monitor the efficacy and pharmacokinetic properties of melatonin. MEL supplements are available in the market for the treatment of sleep disorders or jet lag conditions, aimed to reset the circadian clock. Moreover, through the interaction with MT receptors in different body organs, MEL acts as a central synchronizer regulating a wide range of physiological functions, such as glucose and lipid metabolism. Human clinical trials have shown that MEL may help to reverse/ameliorate certain cardiovascular events.

MEL in the organism acts as an antioxidant, neutralizing a large number of reactive molecules, and indirectly modulates the activity of the endogenous enzymatic antioxidant system. Due to these properties against oxidative stress, as well as the inhibition of different inflammation and apoptotic pathways, MEL has shown neuroprotective effects in vitro and in animal models of Alzheimer’s Disease, Parkinson or Amyotrophic Lateral Sclerosis, among others. These findings show the strong therapeutic potential of this promising molecule to combat against these neurodegenerative pathologies. Finally, the high concentration of melatonin in wine and beer, and the frequency of consumption (without aberrant behaviors) of these two food matrices, make them two excellent vehicles for incorporating melatonin into the diet naturally. Clinical trials are needed to ascertain the impact of the intake of MEL-rich foods for cardio and neurological protection, which can help in the design of new dietary recommendations or functional foods.

## Figures and Tables

**Table 1:** MEL content in different types of wines depending on grape variety, country and harvest. Adapted from Meng et al. [41].

Wine Variety	Country	Vintage	MEL Content (ng/mL)	Reference
Sangiovese Red Wine	Italy	2005	0.4	[37]
Trebbiano White Wine			0.5	
Albana Grappa	Italy	2009	0.3	[42]
Grape Juice			0.5	
Malbec Red Wine	Argentina	2005	0.16	[39]
Cabernet Sauvignon Red Wine			0.24	
Chardonnay White Wine			0.32	
Gropello	Spain	2009	4.1	[40]
Merlot			8.1	
Presses Wines	Spain	2008	74–322	[6]
Racked Wines	Italy		250–423	
Monovarietal Red Wines	Italy	2009	0.14–0.62	[35]
Polyvarietal Red Wines	Italy	2010	0.05–0.31	
White Wine	Italy	2010	0.18	
Dessert Wines	Italy	2007	0–0.31	
Modena Balsamic Vinegars	Italy	2008	0.11–0.13	

**Table 2:** The table summarizes the multiple actions of MEL in reducing oxidative stress. First, the reactive oxygen (ROS) and reactive nitrogen species (RNS) that have been shown to be neutralized by MEL and metabolites that are formed during its antioxidant cascade. Enzymes that impact the redox state of the cell are also identified because they either cause the generation of radicals or metabolize them to inactive products. The former is upregulated while the latter are downregulated by MEL and/or its metabolites. Glutamyl cysteine ligase induces the formation of glutathione, an important intracellular antioxidant. Finally, the list of features that aid MEL in terms of its ability to quench free radicals and reduce oxidative damage is included. Adapted from Limson et al. [57].

Melatonin and Its Metabolits Actions	
**Detoxification of ROS/RNS**	Superoxide anion radical
	Hydrogen peroxide
	Hydroxyl radical
	Peroxynitrite
	Singlet oxygen
	Nitric oxide
	Peroxyl radical
	Alkoxyl radical
	Other organic radicals
**Modulation of redox enzymes**	Superoxide dismutase
	Glutathione peroxidase
	Glutathione reductase
	Glutamyl cysteine ligase
	Cyclo-oxygenase
	Heme-oxygenase
	Nitric-oxide synthase
	Paraoxonase
	Myeloperoxidase
	Lipoxygenase
	Catalase
**Physiological and metabolic features**	Universal distribution in animals and plants
	Endogenous and exogenous availability
	Crosses morphophysiological barriers
	High intracellular concentrations
	Anti-inflamatory
	Binds transition metals
	Synergizes with other antioxidants
	Reduces electron leakage from electron transport chain
	Strenghtens circadian rhythms
	Both receptor-mediated and receptor-independet actions
	Interactions with ubiquitin/proteasome
	Ubiquitous distribution
	Very high levels in the cerebrospinal fluid
